# Is PF-00835231 a Pan-SARS-CoV-2 Mpro Inhibitor? A Comparative Study

**DOI:** 10.3390/molecules26061678

**Published:** 2021-03-17

**Authors:** Mohammad Hassan Baig, Tanuj Sharma, Irfan Ahmad, Mohammed Abohashrh, Mohammad Mahtab Alam, Jae-June Dong

**Affiliations:** 1 Department of Family Medicine, Yonsei University College of Medicine, 50-1 Yonsei-ro, Seodaemun-gu, Seoul 120-752, Korea; mohdhassanbaig@gmail.com (M.H.B.); tanush84@gmail.com (T.S.); 2 Department of Clinical Laboratory Sciences, College of Applied Medical Sciences, King Khalid University, Abha 61421, Saudi Arabia; irfancsmmu@gmail.com; 3 Department of Basic Medical Sciences, College of Applied Medical Sciences, King Khalid University, Abha 61421, Saudi Arabia; mabuhashra@kku.edu.sa (M.A.); mmalam@kku.edu.sa (M.M.A.)

**Keywords:** SARS-CoV-2, main protease, mutants, inhibitors, PF-00835231

## Abstract

The COVID-19 outbreak continues to spread worldwide at a rapid rate. Currently, the absence of any effective antiviral treatment is the major concern for the global population. The reports of the occurrence of various point mutations within the important therapeutic target protein of SARS-CoV-2 has elevated the problem. The SARS-CoV-2 main protease (Mpro) is a major therapeutic target for new antiviral designs. In this study, the efficacy of PF-00835231 was investigated (a Mpro inhibitor under clinical trials) against the Mpro and their reported mutants. Various in silico approaches were used to investigate and compare the efficacy of PF-00835231 and five drugs previously documented to inhibit the Mpro. Our study shows that PF-00835231 is not only effective against the wild type but demonstrates a high affinity against the studied mutants as well.

## 1. Introduction

SARS-CoV-2, the etiological agent of COVID-19, is a pandemic responsible for claiming over a million human lives [1]. More than a thousand drugs are currently in the COVID-19 treatment pipeline; most of which are in the discovery stage and many of these are existing treatments for other conditions currently being evaluated for SARS-CoV-2 [2]. The latest statistics available from ClinicalTrials.gov, a directory of clinical trials funded by the US National Library of Medicine, reveal that 4371 studies are registered worldwide and are growing day by day. Twenty-five trials, including using the most common drug, Hydroxychloroquine, were discontinued [3]. To date only two studies are found in the category of ‘Approved for marketing’, which are expanded access to convalescent plasma and the drug molecule Remdesivir [4,5]. As an urgent consequence of the novelty of SARS-CoV-2 infections and the lack of appropriate drugs, a wide range of techniques and methods are being used to tackle the emerging worldwide COVID-19 pandemic. To date, the therapies suggested are mainly the repurposing of existing drugs chosen for the similarity of their initial indication such as antivirals/antiretrovirals or for the similarity of their mode of action [6,7].

Pp1a and pp1ab polyproteins are formed by SARS-CoV-2 and are processed by two virally encoded cysteine proteases; the main protease (Mpro) and the papain-like protease [8]. In the viral replication process, it is apparent that the action of the Mpro is vital as it processes the p1a/p1ab polyprotein virus proteolytically at more than 10 junctions to produce a set of non-structural proteins (NSPs) essential for virus replication and transcription including RdRp, helicase and the Mpro itself [9]. Of all recognized forms of coronaviruses, the Mpro is the most explored target for drug development because it has almost the same mechanism and active site as MERS-CoV (Middle East Respiratory Syndrome Coronavirus) and SARS-CoV [10,11]. 

Despite the lower mutation rate of the virus, studies have revealed more than 12,000 SARS-CoV-2 genome mutations. Many mutations would not impact the capacity of the virus to transmit or trigger illness because they do not alter the structure of a protein and certain mutations that change proteins are often more likely to damage the virus than to strengthen it [12]. A few vaccines have recently been approved and more than 50 are in different phases of trials. Structural protein mutations that are attacked by the host immune system can impede vaccine efficacy and non-structural protein mutations can develop strains that are resistant to antivirals. Different strains of the virus that are more transmittable than the wild type SARS-CoV-2 have recently been identified in South Africa. In the United Kingdom and several other nations including Europe and Brazil, the extensive spreading of coronavirus variants has placed the world on alert and sparked a new lockdown [13,14]. It picks up minor changes to the genetic code every time the SARS-CoV-2 moves from person to person but researchers are beginning to find variations of how the virus mutates. A major research investment is being made towards the development of new therapeutics or repurposing old drugs as weapons against COVID-19. 

Pfizer has started clinical trials (phase I) with a small molecule PF-07304814 that targets the Mpro of SARS-CoV-2 [15]. It may prove to be the first antiviral drug to target this protein (Mpro) to combat COVID-19. PF-07304814 comprises a phosphate group that renders the compound soluble and cleaves the active antiviral PF-00835231 by alkaline phosphatase enzymes in the tissue [15]. 

**Figure 1:**
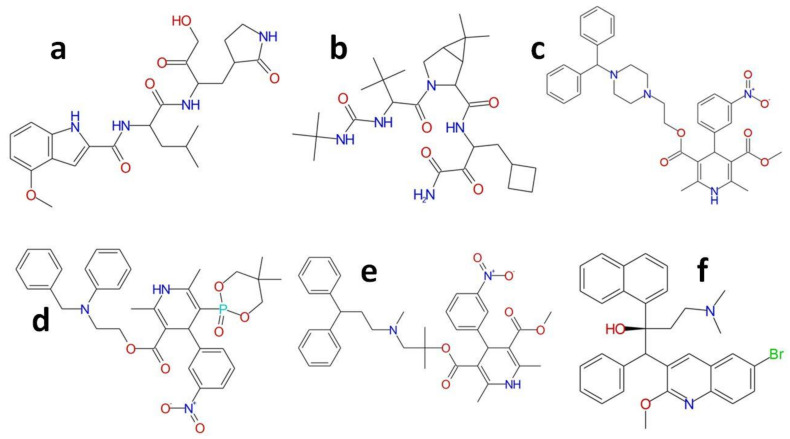
Structure of all of the compounds investigated in this study. (**a**) PF-00835231; (**b**) Boceprevir; (**c**) Manidipine; (**d**) Efonidipine; (**e**) Lercanidipine; (**f**) Bedaquiline.

**Figure 2:**
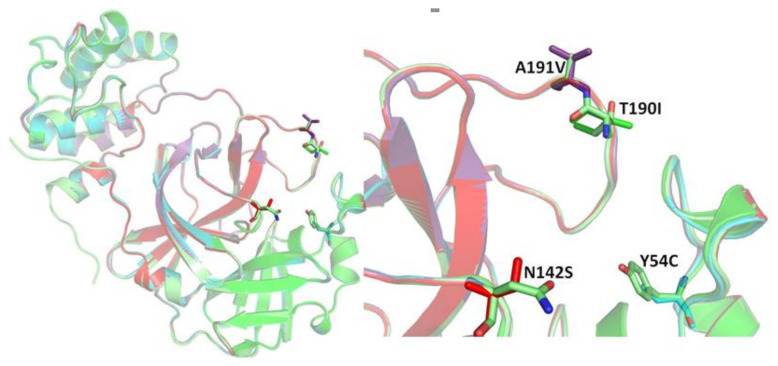
The selected mutant investigated in this study.

Here in this study, we evaluate the binding efficacy of six known inhibitors of the Mpro (Figure 1). The binding efficacy of these inhibitors was measured against the WT and the mutant Mpro (Figure 2). Five drugs (bedaquiline, boceprevir, efonidipine, manidipine and lercanidipine) have been earlier reported to inhibit Mpro activity to below 40 μM [11]. The sixth inhibitor, PF-00835231, is a powerful inhibitor of the SARS-CoV-2 Mpro with sufficient medicinal properties to merit further research as an intravenous COVID-19 therapy [16]. During the 2002–2003 SARS epidemic, PF-00835231 was first discovered by Pfizer chemists to target the SARS-CoV Mpro [16]. Infections petered out, however, and the compound was put on hold along with a collection of other possible coronavirus antivirals. In addition to demonstrating action against two strains of SARS-CoV-2, PF-00835231 was able to kill other coronaviruses in cells as well [15]. PF-00835231 is the active form of PF-07304814 currently being tested (phase 1) in patients with a SARS-CoV-2 infection and mild to moderate symptoms [3,15].

This study involves a state of the art computational evaluation to assess the comparative efficacy of PF-00835231 and other reported inhibitors against the wild type and four reported Mpro mutants. We hypothesize here that PF-00835231 might be less competitive against various SARS-CoV-2 virus mutants; even the results of experimental and clinical studies are still to offer clearer results.

## 2. Result and Discussion

In this study, a comparative analysis of the efficacy of PF-00835231 and five drugs previously documented to inhibit the Mpro (bedaquiline, boceprevir, efonidipine, manidipine and lercanidipine) was performed with the wild type and four reported Mpro mutants (Mutant 1 (Y54C), Mutant 2 (N142S), Mutant 3 (T190I) and Mutant 4 (A191V)).

It was found that in all of the modeled structures, no residues lay in the disallowed region, confirming the significant quality of the structures (Supplementary Figure S1). An ERRAT analysis of all of the structures was also investigated [17]. It was found that the overall structural quality of the modeled structure was very good. A VERIFY_3D [18,19] analysis was also performed and it was found that in all of the modelled structures more than 94% of the residues had an average 3D–1D score > 0.2, proving the great compatibility between the primary sequence to the tertiary structure. Before conducting the molecular docking experiments, the validation of the molecular docking protocol was performed. Different crystal structures of the inhibitor bound SARS-CoV-2 Mpro were retrieved. The binding orientation of the redocked poses was found to be similar to the crystal confirmation of the inhibitor (Supplementary Figure S2 and Table S1). Most of the redocked poses of the inhibitors were found to share a root mean square deviation (RMSD) less than 1 Å (for small molecule inhibitors) than its crystal counterpart (Supplementary Figure S2 and Table S1). 

In order to determine the predictive binding effectiveness of small molecules with receptors, a molecular docking evaluation is usually carried out [20]. Six compounds (PF-00835231, bedaquiline, boceprevir, efonidipine, manidipine and lercanidipine) were minimized and prepared for screening within the active site of the Mpro (WT) and modeled Mpro mutants. In terms of the PLP fitness score using GOLD tools, the binding efficacy score of all six selected compounds was calculated.

**Figure 3:**
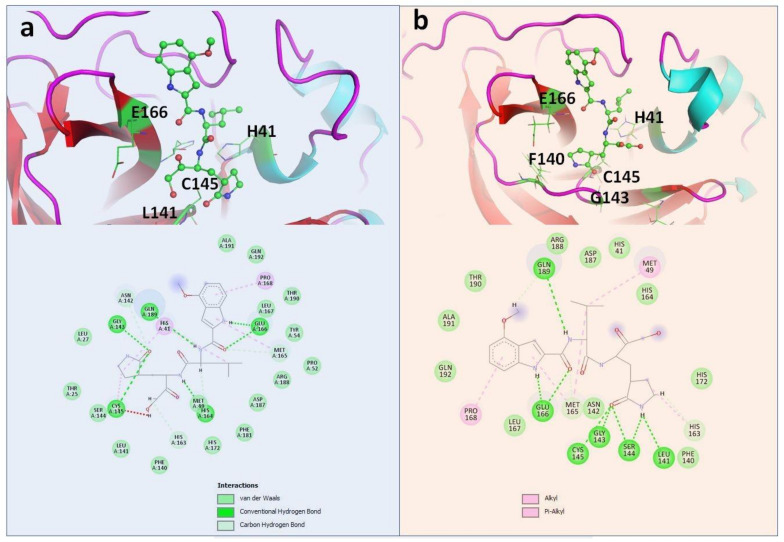
Complex of PF-00835231 within the active site of (**a**) WT; (**b**) Y54C.

**Figure 4:**
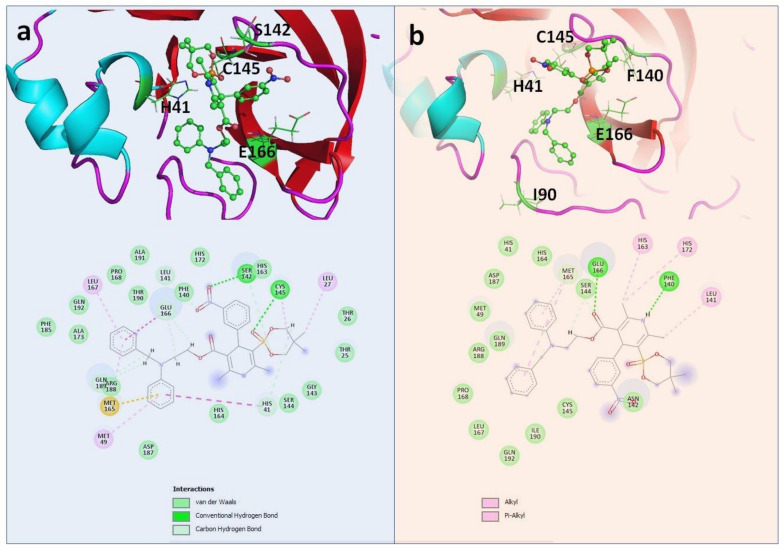
Complex of Efonidipine within the active site of (**a**) N142S; (**b**) T190I.

PF-00835231 was found to be the most effective against the WT Mpro (PLP Fitness score 83.13 (Table 1). This compound was found to be very effective against other selected mutants as well (Table 2, Table 3, Table 4 and Table 5). Compared with other selected compounds, PF-00835231 was found to be the most effective inhibitor against Y54C and A191V (Table 2 and Table 5) (Figure 3) whereas efonidipine was found to be most effective against N142S and T190I (Table 3 and Table 4) (Figure 4). Figure 3 and Figure 4 show the binding of PF-00835231 and efonidipine against the Mpro and the selected mutants. The study also highlighted the important residues playing a crucial role in accommodating the selected compounds within the active site of the Mpro and the mutants. It was also found that the large number of active site residues of the Mpro (WT and mutant) were actively participating in the positioning of all of the molecules. Table 1, Table 2, Table 3, Table 4 and Table 5 represent the details of the interacting residues (amino acid) of the Mpro and mutants interacting with all of the selected molecules. L141, S144, H164, E166, Q189 and Q192 were found to be very prominently involved in making hydrogen bonds with all of the selected compounds (Table 1, Table 2, Table 3, Table 4 and Table 5) (Figure 3 and Figure 4). The crucial role of these residues has been discussed earlier as well [21,22,23]. Other residues found to be playing an important role in the binding were T25, T26, L27, H41, M49, C145, M165, L167, P168, D187 and R188 (Table 1, Table 2, Table 3, Table 4 and Table 5) (Figure 3 and Figure 4 and Supplementary Figures S3–S7). 

Recent studies have shown that all of the compounds (bedaquiline, boceprevir, efonidipine, manidipine and lercanidipine) specified in this study carry the potential to inhibit the Mpro with IC50 values below 40 μM [11]. The binding affinity for PF-00835231 has been reported to be in the nano molar range [15]. Our in depth in silico analysis also found PF-00835231 to be carrying a high affinity against the Mpro (WT) compared with other selected compounds. We also hypothesized that these compounds, including PF-00835231, might prove to be effective against the mutants as well. Boceprevir, which is a protease inhibitor and was originally used to treat hepatitis, has been well studied to carry an inhibitory potential against the Mpro [24,25,26]. This compound was found to carry a very low affinity against the T190I whilst being the most active against the WT. The high binding affinity of this compound against the Mpro (WT) has been reported in several studies [25]. This compound was found to be moderately effective against the WT and the selected mutants. Likewise, other compounds considered in this study showed a moderate affinity against all of the selected mutants. Manidipine [27], which is a calcium channel blocker and is an approved antihypertensive drug, was found to be very effective against the WT. Several studies have reported the potential of manidipine against SARS-CoV-2 [11,28,29]. This compound showed moderate activity against other selected mutants. Lercanidipine [30], another calcium channel blocker and an approved antihypertensive drug, was also found to show moderate activity against all of the selected targets and was most active against the WT and N142S. Bedaquiline [31], another compound considered in this study, is an approved drug for the treatment of active tuberculosis. This compound was found to be moderately active against all of the selected proteins with a maximum binding affinity against N142S and a least binding affinity against A191V. 

**Figure 5:**
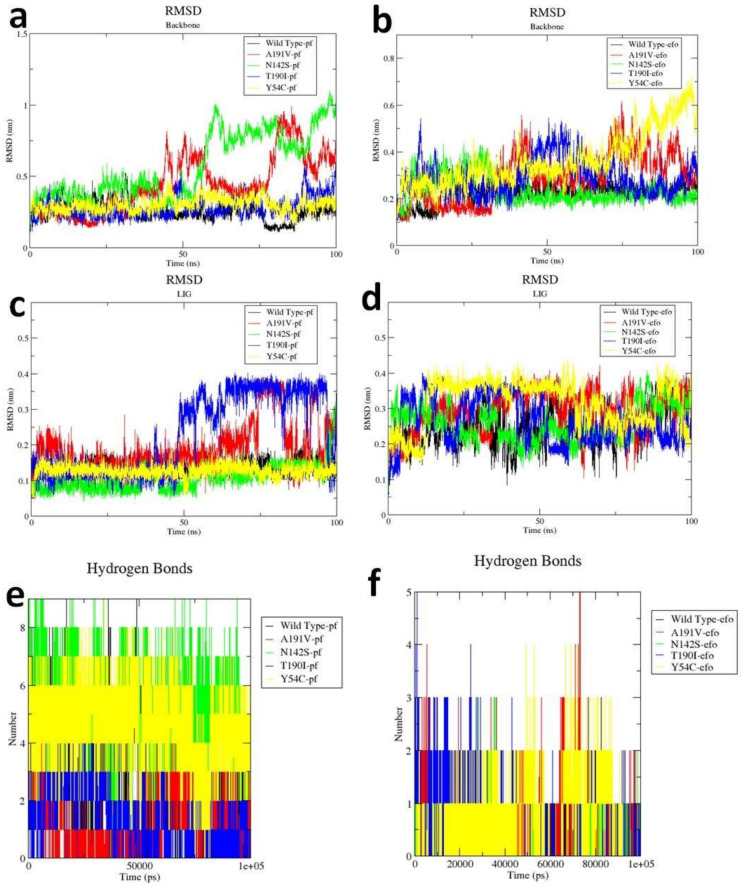
Molecular dynamics results of the PF-07304814 and Efonidipine bound complexes of the WT Mpro (black), Y54C (yellow), N142S (green), T190I (blue) and A1901V (red). The backbone RMSD of the Mpro (WT) and mutants in complex with (**a**) PF-07304814; (**b**) Efonidipine. The ligand RMSD of (**c**) PF-07304814 and (**d**) Efonidipine during the 100 ns. The intermolecular hydrogen bond formations of the (**e**) PF-07304814 and (**f**) Efonidipine bound complex.

Considering the high efficacy of PF-00835231 and Efonidipine against all of the selected proteins, we further studied the structure dynamics of WT and the Mpro mutants in complex with these two inhibitors (Figure 5). Root mean square deviation (RMSD) is a very significant parameter to explore the protein dynamics in terms of conformational changes within the protein structure. The backbone RMSD plot revealed that in the presence of PF-00835231, the structures of the WT, Y54C and T190I mutants were stable throughout the 100 ns simulation while the structures of N142S and A191V indicated fluctuations after 50 ns (Figure 5a). The backbone of WT and N142S was found to be stable in the Efonidipine bound structure (Figure 5b) while other mutants, namely Y54C, A191V and T190I, showed fluctuations in the backbone. Our overall investigation found that Efonidipine caused fewer structural variations in the backbone of WT and N142S while PF-00835231 caused fewer structural variations in the WT, Y54C and T190I mutants. This suggested that the association of PF-00835231 within the active site of the Mpro and its mutant was comparatively more stable. Further, the ligand RMSD plot was also analyzed and it was observed that PF-07304814 was more stable with WT, Y54C and N142S throughout the simulation time period compared with Efonidipine (Figure 5c,d). These analyses further provide a strong support that the PF-07304814 bound complexes were very stable. The Hbond analysis also showed that the PF-00835231 bound complexes comparatively made more hydrogen bonds than the Efonidipine bound complexes (Figure 5e,f). This finding well supports the theory that the stability of PF-07304814 within the active site of the Mpro (WT) and its mutants may be because of the greater number of Hbonds providing the stability to PF-07304814. The overall outcome of this study showed that PF-07304814 could be a very potent inhibitor against the Mpro and its other reported mutants.

## 3. Materials and Methods

### 3.1. Protein Structure Preparation

Here in this study, the crystal structure of the SARS-CoV-2 (COVID-19) Mpro in complex with inhibitor UAW248 was retrieved from the RCSB protein databank (pdb id: 6xbi) [32,33]. The crystal bound inhibitor and other heteroatoms were removed. The structure of the mutants was prepared using the molecular modeling technique. The amino acid sequence of all of the selected mutants (Y54C, N142S, T190I and A191V) were retrieved from the NCBI protein database (GenBank: QJD23268.1, QJC19621.1, QJA16866.1 and QIZ14843.1) [34]. To model the structure of the mutants, the 6xbi was taken as a template. The structures were modelled using the modeler 9.23 [35]. All of the modeled structures were validated using various in silico tools [36,37]. The structure of all of the Mpro mutants was modeled and validated as well. The Ramachandran plot was computed for all of the modeled structures using the PROCHECK module of SAVES [38]. All of the structures were subjected to energy minimization using the steepest descent method for 1000 steps.

### 3.2. Ligand Structure Preparation

The 3D structure of bedaquiline (CID: 5388906), boceprevir (CID: 10324367), efonidipine (CID: 119171), lercanidipine (CID: 65866), manidipine (CID: 4008) and PF-00835231 (CID: 11561899) were retrieved from the PubChem structure database [39]. All of the compounds were energy minimized using the conjugate gradient method for 1000 steps in UCSF Chimera [40].

### 3.3. Redocking: Co-Crystallized Ligand Pose Validation Study

Several crystal structures of the inhibitor bound SARS-CoV-2 Mpro were retrieved from the RCSB protein databank. The inhibitors were separated and were subjected to a redock within the structure of the Mpro using CCDC GOLD [41]. The docked confirmation of the inhibitors within the active site of the Mpro was compared with the crystal orientations.

### 3.4. Virtual Screening

All of the selected compounds were subjected to docking within the active site of WT and selected Mpro mutants using Gold 2.2 (CCDC, Cambridge, UK) [41]. The selection was made based on their PLP fitness score. The complexes were visualized using PyMol [42] and a discovery studio visualizer. 

### 3.5. Molecular Dynamics Simulation

The selected complexes were subjected to a molecular dynamics simulation to investigate the stability of these molecules (in complex with WT and mutant Mpro). The molecular dynamics simulation was performed with GROMACS [43,44]. The complexes of PF-00835231 and efonidipine complexed with the Mpro (WT) and mutants prepared using the molecular docking were considered as a starting point for MD study. Here we used a GROMACS 2020.4 package with a Charmm36 force field to perform the MD simulation [45]. GROMACS is a widely used tool for performing MD simulation studies and its utilization in protein-ligand simulation has been reported in a large number of studies [46,47]. The parameter files for all the ligands were generated using SwissParam (https://www.swissparam.ch/, accessed on 15 January 2021), which is an online tool for generating parameters for the Charmm force field. The complexes were solvated within the dodecahedron box of an explicit TIP3P water model with a 0.1 nm margin between the box walls and solute. Na^+^ or Cl^−^ counterions were added to neutralize the system charge [48,49]. The particle mesh Ewald method (cutoff distance of 0.1 nm) was employed for calculating the long-range electrostatic interactions [50]. The Lennard-Jones 6–12 potential was used for evaluating the van der Waals interactions; for this calculation, the cutoff distance was set to 0.1 nm. The LINCS algorithm was used to constrain the bond lengths while setting the time step to 0.002 pico second [51,52]. Further energy minimization was performed using the steepest descent method for 10,000 steps in order to remove the steric clashes between atoms. The whole system was further subjected for equilibration for 1 nano second (ns). To maintain the system at 300 K and 1 atm, Berendsen weak coupling systems were utilized [53,54]. A Maxwell Boltzmann distribution was used for randomly generating the initial velocities. The final 100 ns production run was performed at 300 K in an NPT ensemble. Furthermore, xmgrace was used to generated graphs (http://plasmagate.weizmann.ac.il, accessed on 15 January 2021); PyMol and VMD were used for further graphical inspections and analysis.

## 4. Conclusions

In conclusion, this study predicted that PF-00835231, which is already being tested to target the SARS-CoV-2 Mpro, may also be potent against the specified Mpro mutants. Notably, PF-00835231 and five other reported antivirals were investigated for comparative inhibitory efficacy in terms of binding potency against WT and the Mpro mutants. PF-00835231 was found to be the most efficient inhibitor of the Y54C and A191V Mpro mutants with a fitness score of 73.17 and 73.61, respectively, relative to the other listed drugs. Based on our research, it is early to determine but hopefully this potential drug PF-00835231 would most certainly be highly effective against the mutant Mpro and could prove to be a sharp weapon in the fight against the COVID-19 pandemic.

## Figures and Tables

**Table 1:** The binding details of all compounds against the WT Mpro.

Protein	Inhibitor	Score	Residues	
			Hydrogen Bond	Pi Interactions
WT	PF-00835231	83.13	G143, C145, H164, E166, Q189	H41, P168
	Manidipine	72.93	F140, N142, E166	H41, L141, M165
	Boceprevir	67.42	N142, E166, Q189	H41, M49, C145
	Lercanidipine	69.73	Q189, Q192	C145, M165, L167, P168
	Efonidipine	71.23		H41, C145, M165, P168, A191
	Bedaquiline	66.92	N142	H41

**Table 2:** The binding details of all compounds against the Mutant 1 (Y54C) Mpro.

Protein	Inhibitor	Score	Residues	
			Hydrogen Bond	Pi Interactions
Mutant 1 (Y54C)	PF-00835231	73.17	L141, G143, S144, C145, E166, Q189	M49, P168
	Manidipine	66.68	T26, S46	C145, M165
	Boceprevir	65.96	T26, G143, C145, E166, Q189	H41, M49, H163, M165
	Lercanidipine	65.28	T26, G143, C145	L27, H41, M165
	Efonidipine	68.23	L141, G143, S144, Q189	L27, C145, H163, P168, H172
	Bedaquiline	63.27	H164	L27, H41, M49, C145

**Table 3:** The binding details of all compounds against the Mutant 2 (N142S) Mpro.

Protein	Inhibitor	Score	Residues	
			Hydrogen Bond	Pi Interaction
Mutant 2 N142S	PF-00835231	76.53	L141, S142, G143, E166, Q189	S144, C145, L167, P168
	Manidipine	68.64	F140, E166	L141, M165
	Boceprevir	65.33	Q189	C44, M49, C145, H163, M165
	Lercanidipine	69.05		L27, M49, C145, H163
	Efonidipine	87.05	S142, C145	L27, M49, M165, L167
	Bedaquiline	68.83	H164	H41, M49, C145, M165

**Table 4:** The binding details of all compounds against the Mutant 3 (T190I) Mpro.

Protein	Inhibitor	Score	Residues	
			Hydrogen Bond	Pi Interactions
Mutant 3 T190I	PF-00835231	70.66	E166, D187, Q189	C44, M49, C145, H163, M165
	Manidipine	63.42	F140, N142	M49, L141, M165
	Boceprevir	60.36	N142	C44, T45, M165
	Lercanidipine	66.96	T26	L27, H41, C44, M49, C145, M165
	Efonidipine	72.25	F140, E166	L141, H163, M165, H172
	Bedaquiline	67.87	E166	H41, C145

**Table 5:** The binding details of all compounds against the Mutant 4 (A191V) Mpro.

Protein	Inhibitor	Score	Residues	
			Hydrogen Bond	Pi Interaction
Mutant 4 A191V	PF-00835231	73.61	L141, G143, S144, C145, Q189	M165, P168, H172, V191
	Manidipine	65.95	T25, S46, N142	M165
	Boceprevir	62.08	S46, Q189	C44, M49, M165
	Lercanidipine	67.93	C44, S46, N142	H41, M49, C145, M165
	Efonidipine	71.01	F140	L141, H163, M165, P168
	Bedaquiline	59.99	N142	H41, C145, M165

## Data Availability

Data is available within the article.

## Supplementary Materials

The following are available online: Figure S1: Validation of the modeled structures of mutants (a) Y54C (b) N142S (c) T190I (d) A191V, Figure S2: The superimposed structure of crystal pose (blue) of inhibitor and redocked pose of (a) X47 (green) (pdb id: 6wco), (b) X77 (yellow) (pdb id: 6w63), and (c) ADRAFINIL (red) (pdb id: 7ans) within the binding site of Mpro, Figure S3: Complex of (a) PF-00835231 (b) Boceprevir (c) Manidipine (d) Efonidipine (e) Lercanidipine (f) Bedaquiline within the active site of WT, Figure S4: Complex of all (a) PF-00835231 (b) Boceprevir (c) Manidipine (d) Efonidipine (e) Lercanidipine (f) Bedaquiline within the active site of Y54C, Figure S5: Complex of all (a) PF-00835231 (b) Boceprevir (c) Manidipine (d) Efonidipine (e) Lercanidipine (f) Bedaquiline within the active site of N142S, Figure S6: Complex of all (a) PF-00835231 (b) Boceprevir (c) Manidipine (d) Efonidipine (e) Lercanidipine (f) Bedaquiline within the active site of T190I, Table S1: The inhibitor bound crystal structure of SARS-CoV-2 Mpro considered for the validation of docking protocol.

## Sample Availability

Samples of the compounds are not available from the authors.
