# Supplementary material for: Is PF-00835231 a Pan-SARS-CoV-2 Mpro Inhibitor? A Comparative Study
Source: Molecules. 2021 Mar 17;26(6):1678. doi: 10.3390/molecules26061678 (PMC8002701; doi:10.3390/molecules26061678)
Supplement: Supplementary file 1 [file molecules-26-01678-s001.pdf]

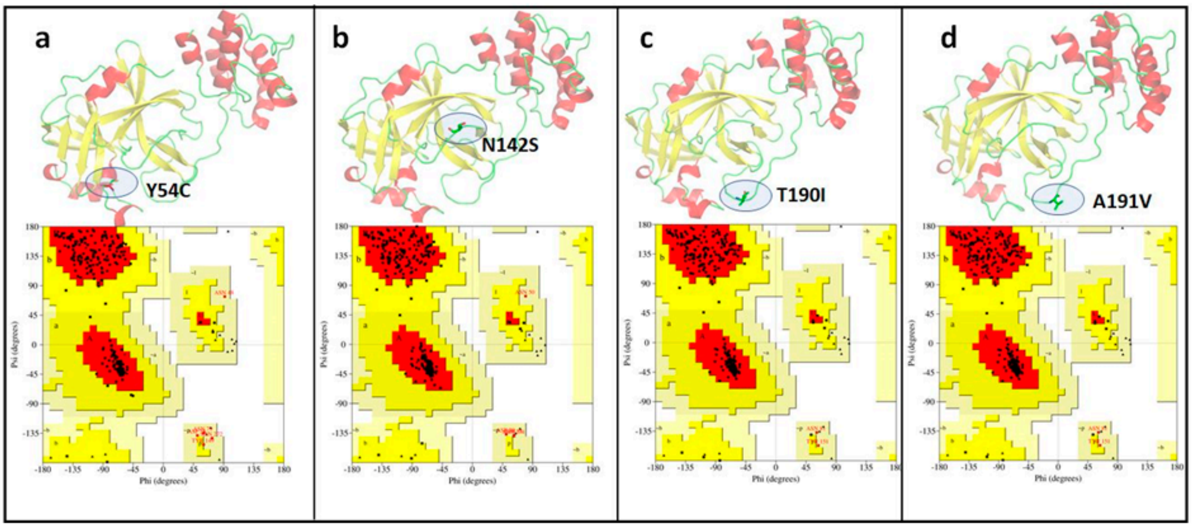

Figure S1. Validation of the modeled structures of mutants (**a**) Y54C (**b**) N142S (**c**) T190I (d) A191V.

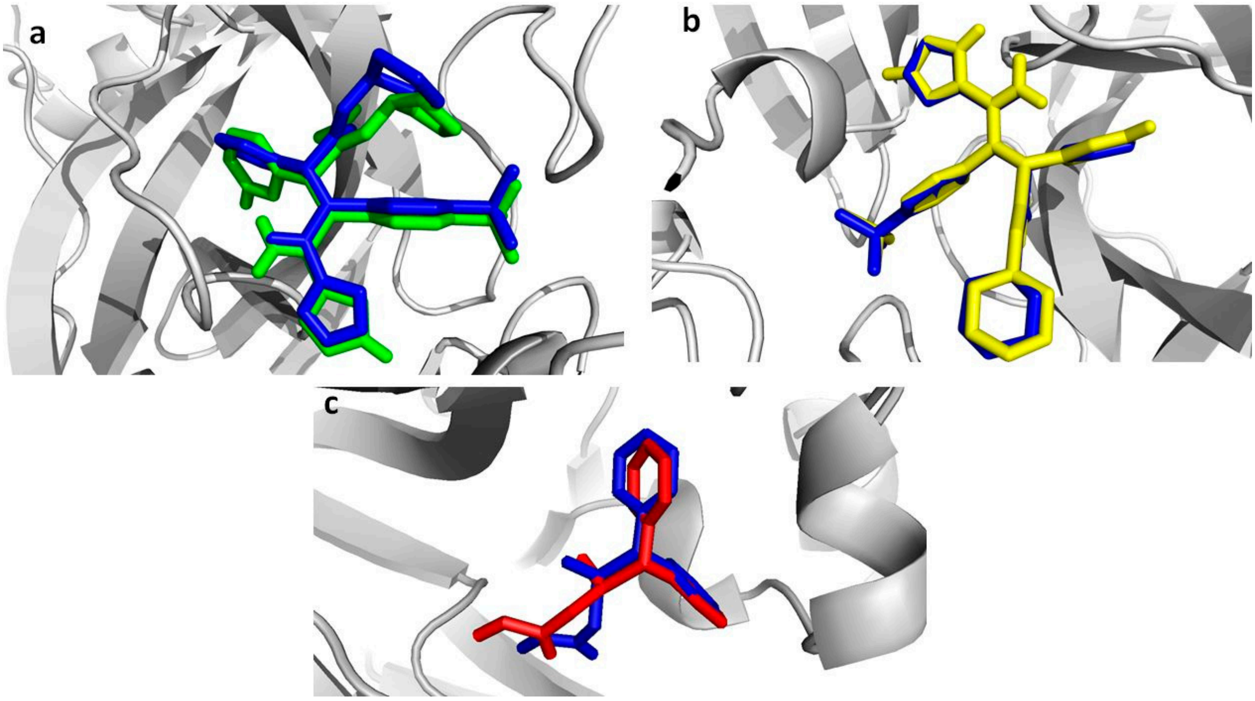

Figure S2. The superimposed structure of crystal pose (blue) of inhibitor and redocked pose of (**a**) X47 (green) (pdb id: 6wco), (**b**) X77 (yellow) (pdb id: 6w63), and (**c**) ADRAFINIL (red) (pdb id: 7ans) within the binding site of Mpro.

Table S1. The inhibitor bound crystal structure of SARS-CoV-2 Mpro considered for the validation of docking protocol.

Pdb id	Detail	RMSD (Å) original and re-docked pose	Class of molecule
6wco	STRUCTURE OF SARS MAIN PROTEASE BOUND TO INHIBITOR X47	0.9792	Small molecule inhibitor
6w63	STRUCTURE OF COVID-19 MAIN PROTEASE BOUND TO POTENT BROAD-SPECTRUM NON-COVALENT INHIBITOR X77	1.2026	Small molecule inhibitor
7ans	STRUCTURE OF SARS-COV-2 MAIN PROTEASE BOUND TO ADRAFINIL	0.8961	Small molecule inhibitor

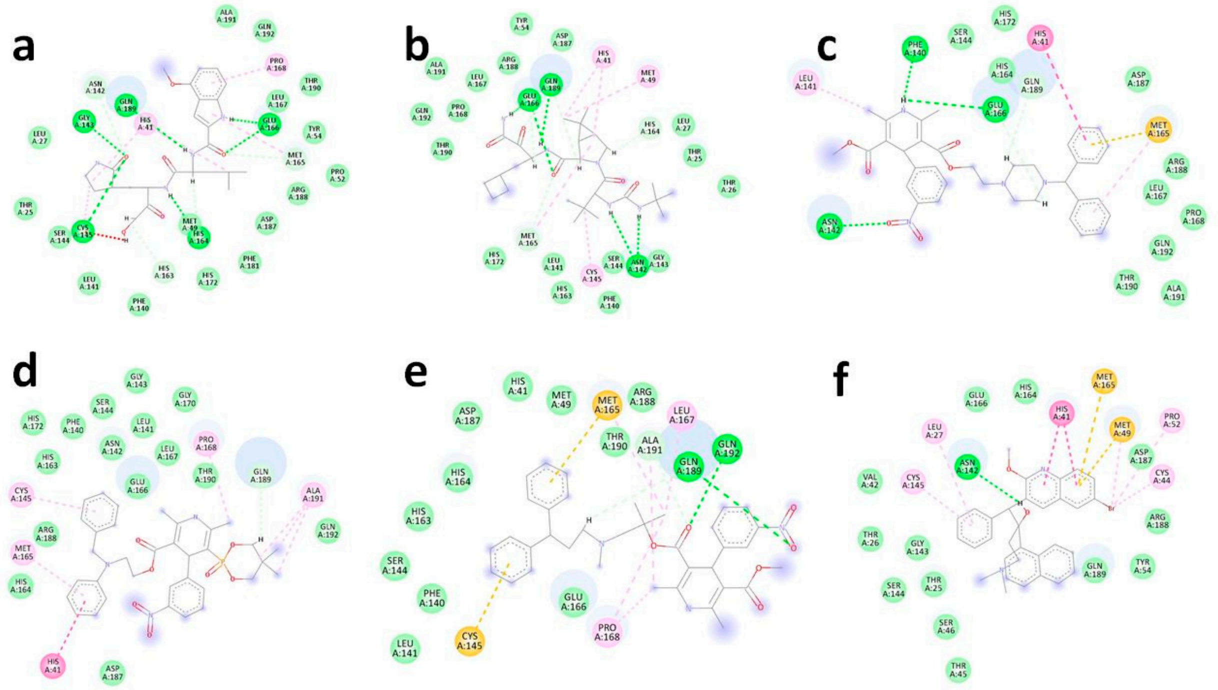

Figure S3. Complex of (**a**) PF-00835231 (**b**) Boceprevir (**c**) Manidipine (**d**) Efonidipine (**e**) Lercanidipine (**f**) Bedaquiline within the active site of WT.

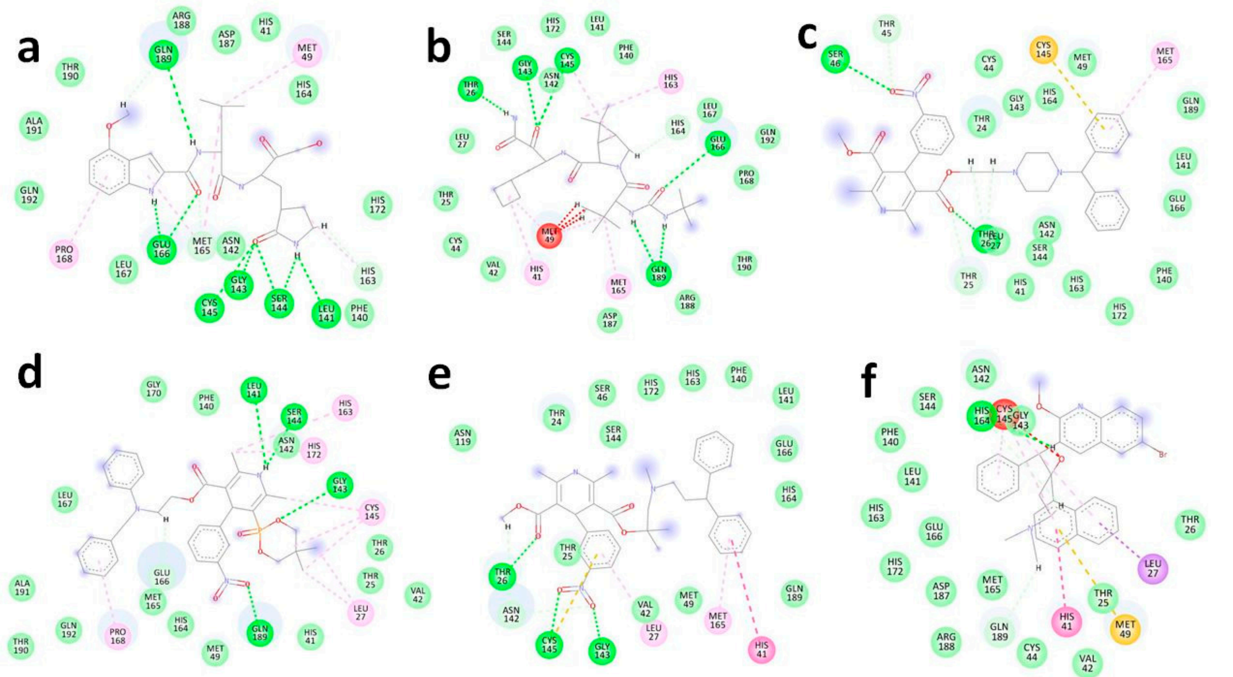

Figure S4. Complex of all (**a**) PF-00835231 (**b**) Boceprevir (**c**) Manidipine (**d**) Efonidipine (**e**) Lercanidipine (**f**) Bedaquiline within the active site of Y54C.

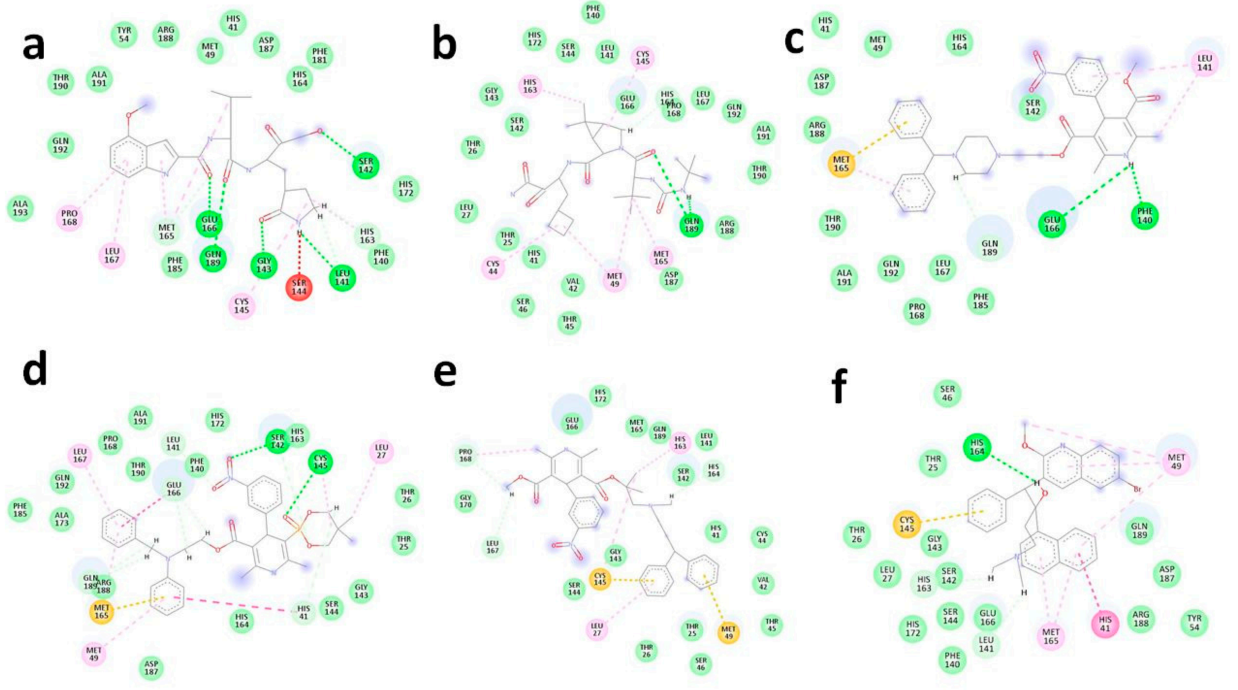

Figure S5. Complex of all (**a**) PF-00835231 (**b**) Boceprevir (**c**) Manidipine (**d**) Efonidipine (**e**) Lercanidipine (**f**) Bedaquiline within the active site of N142S.

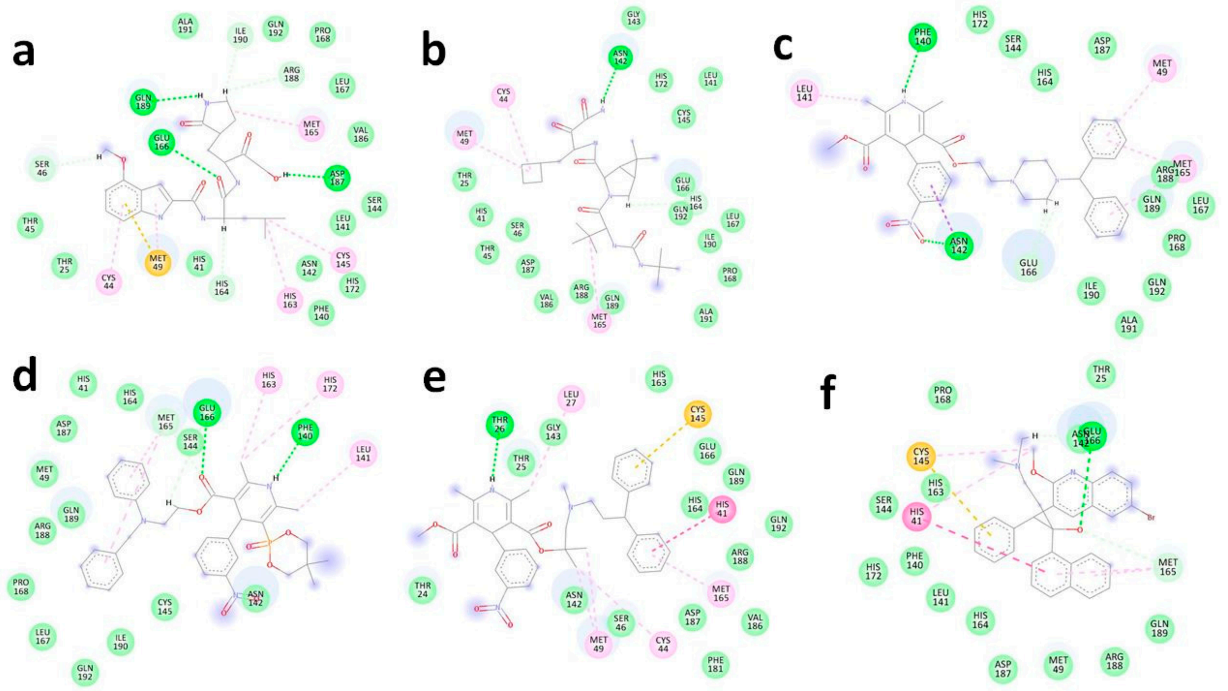

Figure S6. Complex of all (**a**) PF-00835231 (**b**) Boceprevir (**c**) Manidipine (**d**) Efonidipine (**e**) Lercanidipine (**f**) Bedaquiline within the active site of T190I.

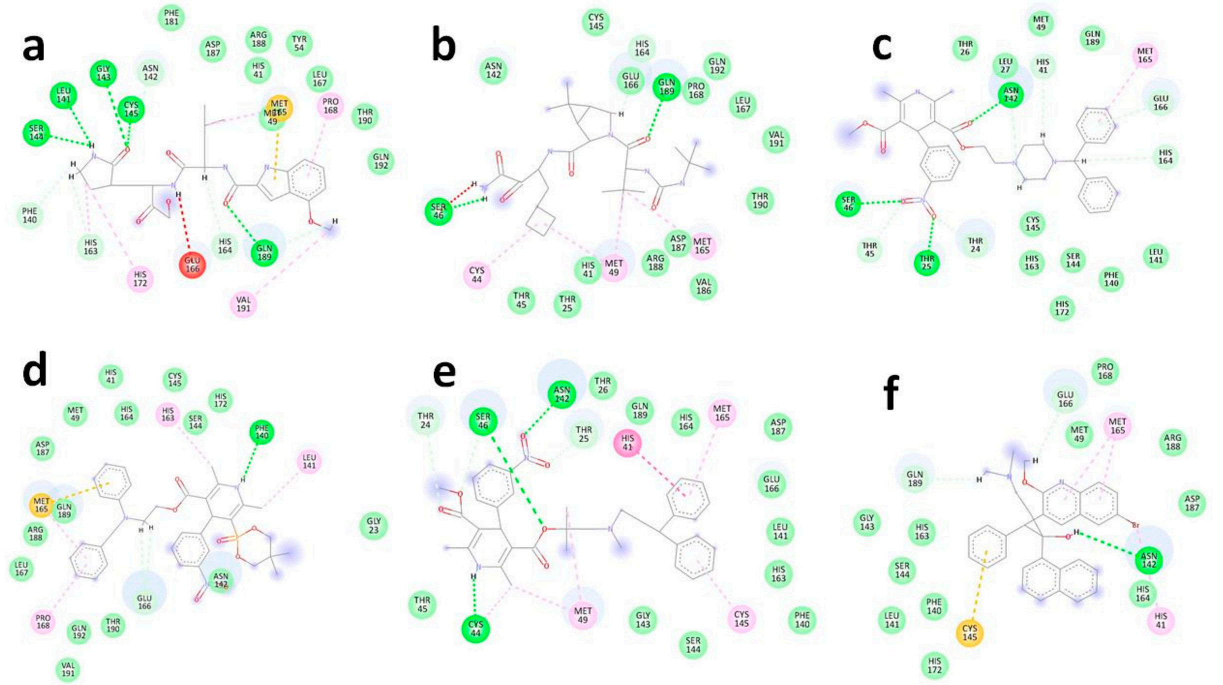

Figure S7. Complex of all (**a**) PF-00835231 (**b**) Boceprevir (**c**) Manidipine (**d**) Efonidipine (**e**) Lercanidipine (**f**) Bedaquiline within the active site of A191V.
